# Comparing Basic Life Support Serious Gaming Scores With Hands-on Training Platform Performance Scores: Pilot Simulation Study for Basic Life Support Training

**DOI:** 10.2196/24166

**Published:** 2020-11-25

**Authors:** Mehmet Emin Aksoy

**Affiliations:** 1 Acibadem Mehmet Ali Aydınlar University Department Biomedical Device Technology CASE (Center of Advanced Simulation and Education) Istanbul Turkey

**Keywords:** serious gaming, medical simulation, basic life support

## Abstract

**Background:**

Serious games enrich simulation-based health care trainings and improve knowledge, skills, and self-confidence of learners while entertaining them.

**Objective:**

A platform which can combine performance data from a basic life support (BLS) serious game app and hands-on data based on the same scoring system is not available in the market. The aim of this study was to create such a platform and investigate whether performance evaluation of BLS trainings would be more objective compared to conventional Objective Structured Clinical Examination (OSCE) examinations if these evaluations were carried out with the platform which combines OSCE scoring criteria with sensor data retrieved from the simulator’s sensors.

**Methods:**

Participants were 25 volunteers (11 men [44.0%] and 14 [56.0] women) among Acıbadem Mehmet Ali Aydınlar University students without prior knowledge of the BLS protocol. A serious game module has been created for teaching learners the European Resuscitation Council Basic Life Support 2015 protocol. A second module called the hands-on module was designed for educators. This module includes a checklist used for BLS OSCE examinations and can retrieve sensor data such as compression depth, compression frequency, and ventilation volume from the manikin (CPR Lilly; 3B Scientific GmbH) via Bluetooth. Data retrieved from the sensors of the manikin enable educators to evaluate learners in a more objective way. Performance data retrieved from the serious gaming module have been combined with the results of the hands-on module. Data acquired from the hands-on module have also been compared with the results of conventional OSCE scores of the participants, which were obtained by watching the videos of the same trainings.

**Results:**

Participants were considered successful in the game if they scored 80/100 or above. Overall, participants scored 80 or above in an average of 1.4 (SD 0.65) trials. The average BLS serious game score was 88.3/100 (SD 5.17) and hands-on average score was 70.7/100 (SD 17.3), whereas the OSCE average score was 84.4/100 (SD 12.9). There was no statistically significant correlation between success on trials (score ≥80/100), serious game, hands-on training app, and OSCE scores (Spearman rho test, *P*>.05). The mean BLS serious game score of the participants was 88.3/100 (SD 5.17), whereas their mean hands-on training app score was 70.7/100 (SD 17.3) and OSCE score was 84.4/100 (SD 12.9).

**Conclusions:**

Although scoring criteria for OSCE and hands-on training app were identical, OSCE scores were 17% higher than hands-on training app scores. After analyzing the difference of scores between hands-on training app and OSCE, it has been revealed that these differences originate from scoring parameters such as compression depth, compression frequency, and ventilation volume. These data suggest that evaluation of BLS trainings would be more objective if these evaluations were carried out with the modality, which combines visual OSCE scoring criteria with sensor data retrieved from the simulator’s sensors.

**Trial Registration:**

ClinicalTrials.gov NCT04533893; https://clinicaltrials.gov/ct2/show/NCT04533893

## Introduction

The effectiveness of serious gaming has been demonstrated primarily by studies of higher education, government, corporate, and military environments. Serious gaming and medical simulation techniques, which are 2 complementary modalities for training of tomorrow’s physicians, have gained popularity in the past decade. Olszewski and Wolbrink [1] emphasize that serious games are increasingly being used for medical education. Blended learning methodology combines serious gaming with medical simulation programs and enhances the efficiency and effectiveness of training programs [2]. E-learning by using serious gaming refers to the use of internet technologies to deliver an interactive educational content that enhances knowledge and performance [3,4]. Prensky [5] concluded that game technologies can produce a great deal of learning and positive effects such as quickly mastering and applying new skills and information, thinking strategically, and persisting to solve difficult problems. David and Michael [6] described serious game methodology as using game-based simulations that do not have entertainment, enjoyment, or fun as their primary purpose.

Nowadays, after providing knowledge using serious game–based apps, simulation-based trainings and Objective Structured Clinical Examination (OSCE) are used to assess hands-on training performance of trainees [7,8].

Basic life support (BLS) is the most frequently organized training course for both medical and nonmedical trainees. Depending on the country, BLS certifications have to be renewed on a yearly basis. Smith et al [9] inspected the decline of BLS skills retention in 3, 6, 9, and 12 months. In BLS, even after 3 months, test scores of trainees decreased by 33.3% compared to their posttraining test scores. Decrease in knowledge and skills are more dramatic after 6, 9, and 12 months [9]. Therefore, BLS providers need more frequent refresher training to improve and maintain their knowledge, skills, and self-confidence.

Depending on these facts, a multiplatform-compatible interactive serious game module was created in our simulation center to teach adult BLS Protocol based on the European Resuscitation Council (ERC) 2015 Criteria [10]. As serious gaming performance analysis is only used for knowledge domain assessment, effectivity of BLS trainings also has to be assessed by observing hands-on performance of learners. Therefore, it has been decided to develop a tablet-based app for educators, which is capable of retrieving sensor data such as compression depth, compression frequency, ventilation volume from the manikin via Bluetooth and includes OSCE criteria for BLS training. Sensor data retrieved from the manikin enable educators to evaluate learners in a more objective way. Both performance data from the serious gaming module and hands-on training app can be stored on the same learning management system (LMS), which allows educators to track and compare performances on individual basis. There is no available platform in the market which can combine performance data from the BLS serious game app and hands-on data based on the same scoring system.

As OSCEs are mainly based on observation of the simulation-based trainings, this new platform, which combines visual OSCE scoring criteria with sensor data retrieved from the simulator’s sensors, would be able to provide more objective and precise scores for BLS trainings. The hypothesis of this study is that whether performance evaluation of BLS trainings would be more objective compared to conventional OSCEs, if these evaluations were carried out with the modality which combines visual OSCE scoring criteria with sensor data retrieved from the simulator’s sensors.

## Methods

### Recruitment

After obtaining approval from the Acıbadem Mehmet Ali Aydınlar University Ethics Committee, participants were chosen among volunteers and asked to fill and sign consent forms. The participants of this study included 25 volunteers among Acıbadem Mehmet Ali Aydınlar University students without prior knowledge of BLS protocol; 11 participants (44%) were male and 14 (56%) were female. Volunteers with prior BLS training were excluded from the study.

### Study Design

**Figure 1:**
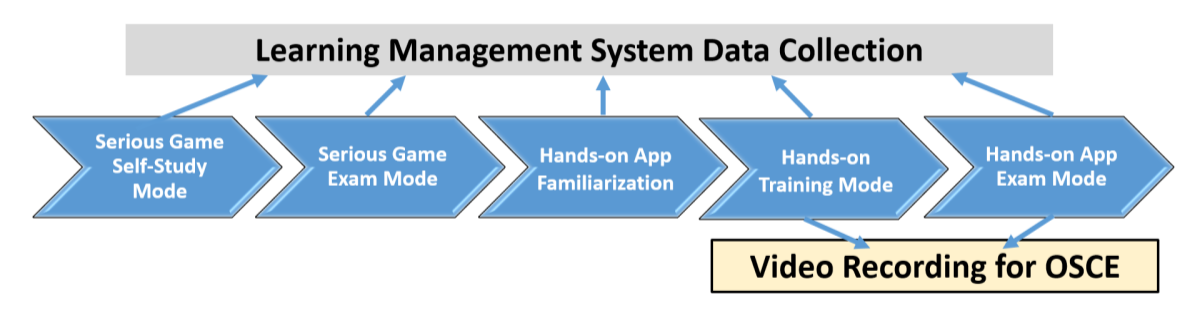
Design of the study flow.

After a brief introduction of the serious gaming module, the participants were asked to log into the system with their own usernames and passwords. After completing the training mode of the serious game module, they were asked to choose the self-test mode of the module. The participants’ number of attempts was not limited. Because of the COVID-19 pandemic, the participants were asked to practice their hands-on skills in the simulation center under the supervision of educators on the same day in order to minimize the risk of contamination. The new hands-on training app, which combines OSCE scoring criteria with sensor data retrieved from the simulator’s sensors, was used for hands-on performance evaluation. After familiarization with the system using the self-training mode, the participants were asked to use the BLS hands-on training app with the simulator under the supervision of the educator. The simulation sessions were also recorded in order to use these recordings for conventional OSCE performance evaluation. Conventional OSCE scores of participants were obtained by watching the recorded sessions of BLS trainings. The study design is shown in Figure 1.

### Serious Game–Based BLS Training App

**Figure 2:**
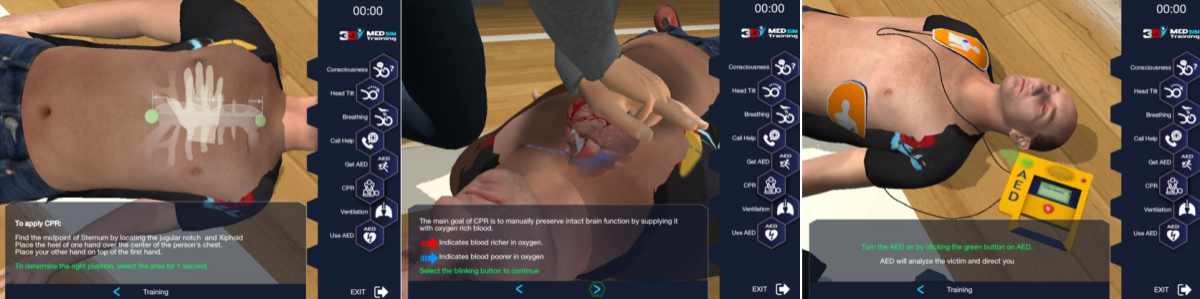
Screen captures from the Basic Life Support Serious Game Module.

The tablet-based serious game module is compatible with 2015 Basic Life Support Algorithm of the ERC and includes an LMS, a learning record store (LRS), and a 3D visualization engine (VE) [11,12]. LMS and LRS enable to store users’ credentials in a shared database. Through the interaction between LRS and VE every action performed by the user can be followed on the VE side and experience API (xAPI) calls corresponding to each action can be made to the LRS [12,13]. During serious game play, whenever the user has entered a predefined area or whenever the user has triggered some kind of predefined interaction with a significant object that is part of the virtual environment, an xAPI event is automatically generated [12-14]. In order to provide this interaction, a software library has been developed as a unity extension. The main function of this library is to perform the necessary xAPI web service calls, to the LRS servers, over the HTTP protocol. All actions, which may need to be performed by the user as part of an education scenario, are defined in terms of the actor, verb, and object basic parameters. As these actions are performed by the user, the library automatically issues the xAPI calls corresponding to each action, and the LRS keeps track of each significant action through these calls [12-14]. In addition to actions directly performed by the user, it is also possible to collect xAPI calls automatically for actions that have not been performed within a given time limit. The library also implements the security and user authentication features necessary for keeping proper records. For this purpose, the basic access authentication method which is part of the HTTP protocol standard is used. The serious game study was named as “3D SIM Training Basic Life Support.” 3D SIM Training Basic Life Support is a multilingual serious game app with 3D and interactive features, based on ERC 2015 Guidelines. The app consists of a training mode, a self-test mode, and a review mode enabling users to track their previous records. The training module guides the user step by step through a scenario. The user has to go through a training mode followed by the self-test mode. The user is not in the passive learner mode but has an active role in the game. The user is first instructed about the correct actions to be taken and then interacts with the 3D software while playing the rescuer role interactively. All main steps of the ERC 2015 Adult BLS algorithm are covered in the BLS Serious Game app. The user is expected to finish 1 stage correctly before continuing with the next stage. The app is developed as a 3D real-time game enabling the user to interact and view the scene from different angles. A 3D animation-like chest compression timing clarifies some of the steps within the BLS algorithm and gives the trainees the feeling of a chest compression frequency between 100 and 120 compressions/minute. Screen captures from the Adult BLS Module are presented in Figure 2. The animation of blood flow during CPR informs the users about the aim of the chest compression. The right locations for checking carotid pulse, chest compressions, and automated external defibrillator pad locations are also indicated during the self-training mode.

**Figure 3:**
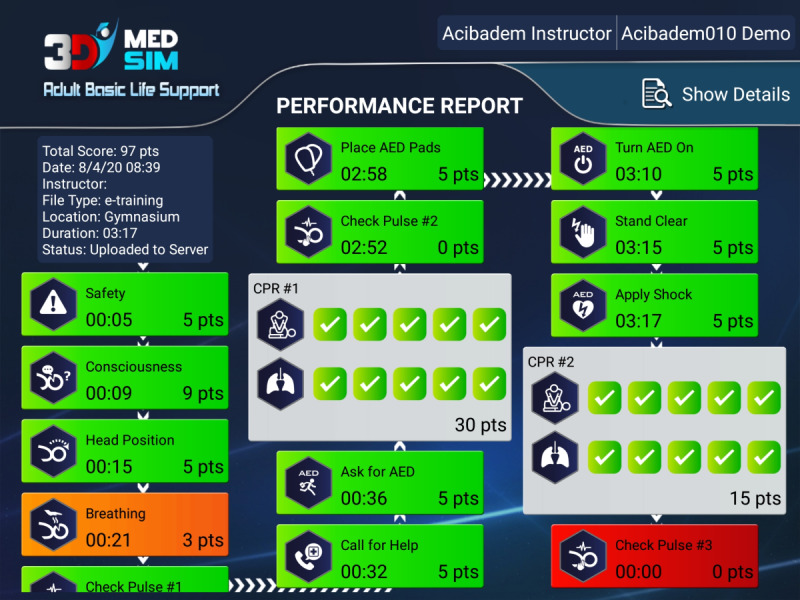
Performance report of BLS serious game app.

Once the user successfully finishes the training module, he/she has to complete the self-test module. Unlike the training module, users are expected to perform the correct steps with correct timing without hints or instructions to follow in the self-training mode. The SCORM (Sharable Content Object Reference Model)-xAPI standard has been used for this serious game module due to its advantages such as supporting a variety of content types, simplicity for implementing, and supporting offline or disconnected scenarios. Because of these advantages, the SCORM-xAPI standard has been chosen as the methodology to track the digital learning content [4,15]. Because of its advanced features, x-API can be considered as a replacement for previous e-learning standards. In this serious game module, all actions and their timings are tracked using the SCORM-xAPI standard and the outcome of the “game” depends on the user’s success. At the end of the scenario, the user is presented with an easily understandable performance chart. The user’s performance can also be reported to an internet-based server to create test results for offline evaluation. The reported performance summary incorporates all the expected steps and also shows if they have been performed in the right order and correct timing as shown in Figure 3.

Each step of the user’s performance is evaluated with 3 different colors. Green symbolizes that the step has been successfully applied in appropriate time. Orange symbolizes that the step has been successfully applied but timing was wrong. Red symbolizes that the step has not been applied at all. A virtual 3D simulation environment is developed for training of medical staff for various medical processes. Integration and coordination of 3 main parts, including 3D VE, LMS, and LRS, were necessary for the functionality of the whole system. Technical suitability, wide platform support (iOS, Android, Windows, Linux, and web), large user database, effective support, and favorable licensing conditions are the parameters for selection of a 3D VE in an educational simulation project [16-19]. Among the criteria mentioned above, technical suitability deserves a more detailed explanation and covers all possible technical requirements specific to the implementation of a 3D education and simulation app integrated with an LMS. This consideration arises because modern VEs are not strictly limited to visualization anymore. Essentially, they are contained within larger frameworks which provide the functionality required for developing entire apps [16-19]. In general, currently available engines have been designed, first and foremost, with the needs of game developers in mind [20]. For realization of this study, performances of a number of 3D visualization and game engines have been evaluated and compared. After evaluating available game engine products, the Unity game engine has been found the most suitable for this study, because it covers all of the criteria outlined above [21]. Owing to the highly extensible architecture of the Unity engine, the users greatly extend the capabilities of the engine by creating a large number of useful and affordable extensions [21].

### Learning Management System (LMS)

LMS is a software designed for managing user learning interventions and delivering learning content to the learners [13,14,22]. LMS is the key for flexible, interactive, multimedia, and decentralized teaching [12-14]. The advantages of using LMS for the educator and learner has been revealed in several studies [23,24]. Therefore, a custom-designed LMS has been integrated to the serious game module in this project.

### Learning Record Store

LRS is a cloud-based service that deals with learning information storage and retrieval of learning information [13,25]. For the LRS, the open source reference LRS implementation, provided by advanced distributed learning, has been selected for this project [26].

### Scoring System

A successful serious game project requires the LMS, the LRS, and the 3D VE to operate in harmony. For this purpose, a software library has been developed as a Unity extension. The main function of this library is to record the track of every action during the game through xAPI calls [12,13,27]. In addition to the actions directly performed by the user, it is also possible to have xAPI calls issued automatically for actions that have not been performed within a given time limit. The library also implements the security and user authentication features necessary for keeping proper records. For this purpose, the basic access authentication method which is part of the HTTP standard was used. Login usernames and passwords were sent to the participants by email after they have downloaded the serious game from iTunes or Google Play. The scores of the participants were stored with the help of the LMS. The participants’ scores were downloaded from the LMS of the serious game module and converted to an MS Excel file format. The same scoring criteria were used for BLS serious gaming app, hands-on app, and OSCE performance analysis as shown in Table 1.

**Table 1:** Score comparisons according to gender.

Condition	Scoring		
	BLS^a^ serious game	Hands-on app with manikin	OSCE^b^
Check Safety	5	5	5
Check Consciousness	9	9	9
Head Tilt	5	5	5
Check Breathing	6	6	6
Call for Help	5	5	5
Get AED^c^	5	5	5
Check Carotid Pulse	0	0	0
1st CPR30^d^	4	4	4
1st Ventilation	2	2	2
2nd CPR30	4	4	4
2nd Ventilation	2	2	2
3rd CPR30	4	4	4
3rd Ventilation	2	2	2
4th CPR30	4	4	4
4th Ventilation	2	2	2
5th CPR30	4	4	4
5th Ventilation	2	2	2
2nd Carotid Pulse	0	0	0
AED Use Pad	5	5	5
AED On Off	5	5	5
Stand Clear	5	5	5
AED Shock	5	5	5
Final CPR	15	15	15
Check Rhythm	0	0	0
Total score	100	100	100

^a^BLS: basic life support.

^b^OSCE: Objective Structured Clinical Examination.

^c^AED: automated external defibrillator.

^d^CPR: cardiopulmonary resuscitation.

### Hands-On Module

**Figure 4:**
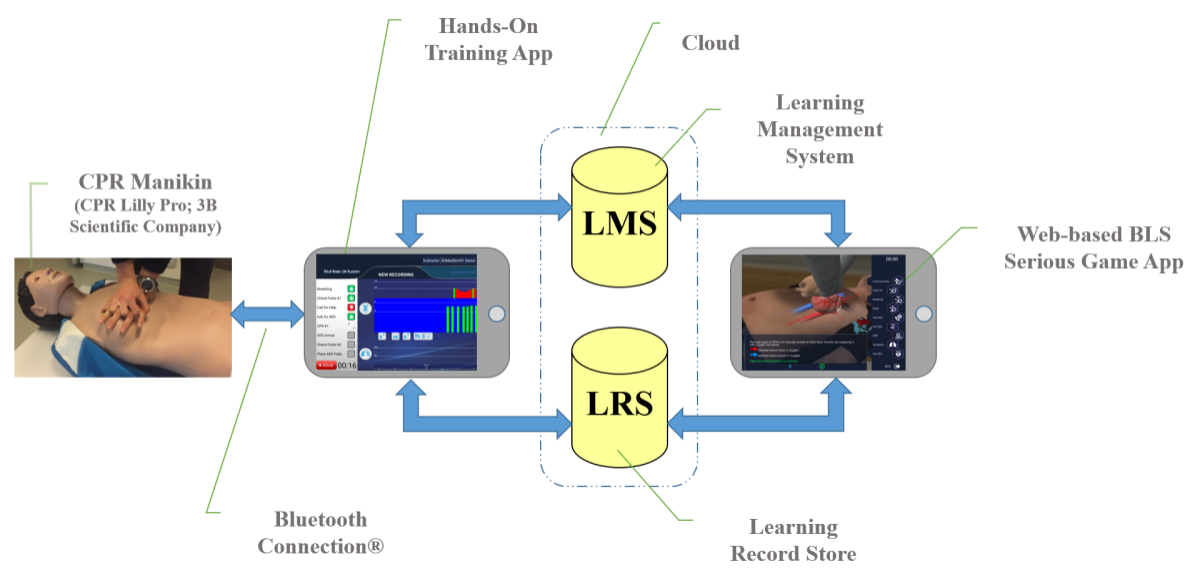
System architecture of the platform.

Hands-on module is a platform developed for BLS training featuring hands-on training. The system is built around an Android app, which communicates with a commercially available BLS manikin (CPR Lilly; 3B Scientific GmbH). The system allows learners to practice, to track their progress using cloud services, and allows educators to conduct BLS examinations with OSCE criteria combined with sensor data obtained from the manikin via Bluetooth. Data acquired from the hands-on module are also stored by the same LMS, which is also used by the BLS serious gaming app. In this way, results from the BLS serious gaming app and hands-on module are stored at individual level by the LMS, so that each learner’s performance data at the knowledge level and hands-on skill level can be tracked by the educator. The system architecture is shown in Figure 4. The system comprises 5 parts as shown in Figure 4.

**Figure 5:**
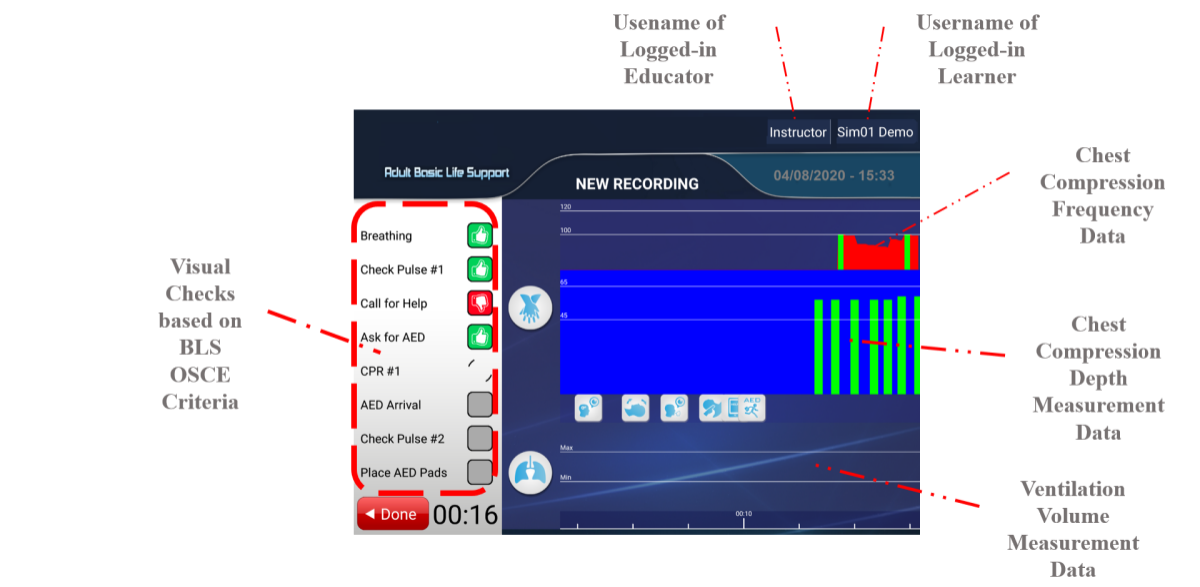
Screen capture of the Hands-on app.

The LRS stores the progress of the learners’ hands-on training sessions in a detailed step-by-step fashion. This information is used to provide the learners with a history of past sessions as well as an up-to-date report card. LMS also accesses the LRS to generate reports for instructors. The hands-on BLS training platform supports any xAPI-compliant third-party LRS. xAPI is an e-learning specification that makes it possible to collect data within online and offline training activities. It is a shared format for receiving and sending learning data between multiple systems. The app provides the learners and instructors with a visual interface to access the past records and current report card. It also communicates with the manikin to record BLS sessions. The manikin contains internal sensors, which provide measurements for chest compression depth and ventilation volume. These measurements can be provided to Bluetooth clients in real time. An instructor must first login to the app to use the system. The app validates the instructor’s credentials against the LMS. Learners must use their credentials to allow the instructor access their data. The app confirms with LMS that the 2 users are entitled to each other for the BLS training course. A screen capture of the hands-on app is shown on Figure 5. The hands-on app combines visual checks based on BLS OSCE criteria and live sensor values for chest compression depth and frequency and ventilation volume obtained from the BLS manikin’s sensors via Bluetooth.

**Figure 6:**
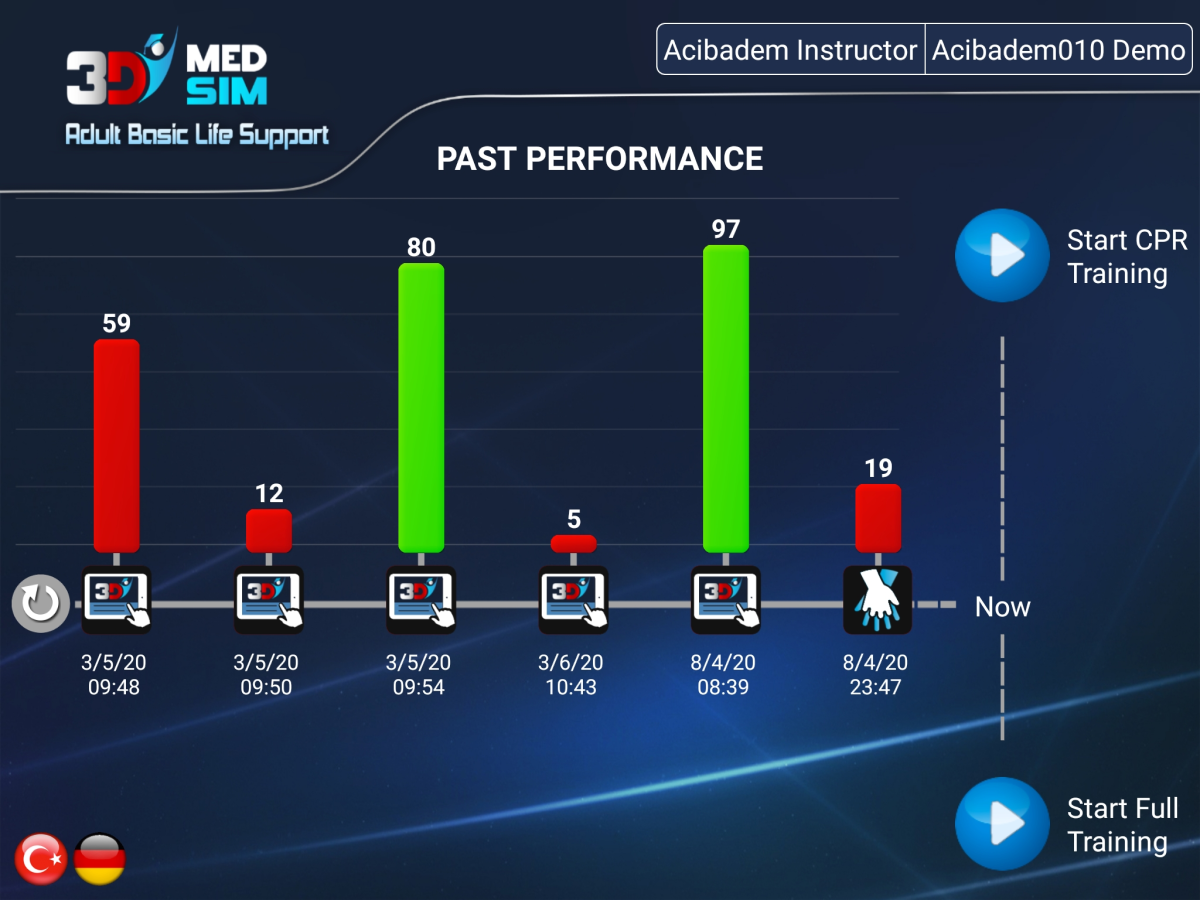
Hands-on app past performance screen.

The app obtains LRS access parameters and uses them to download records of past BLS sessions of the learners, along with their scores for these sessions and previous BLS serious game scores as shown in Figure 6.

**Figure 7:**
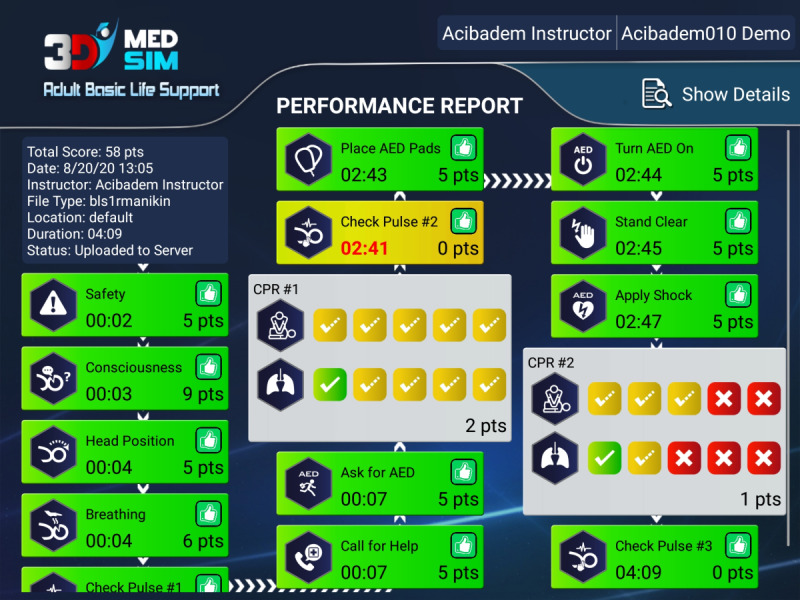
Performance report screen hands-on app.

Choosing to review a past session displays a report card for the session, which contains a score and the BLS process broken down into steps. Each step marked with a score component, timing, evaluation, and rationale as shown in Figure 7.

**Figure 8:**
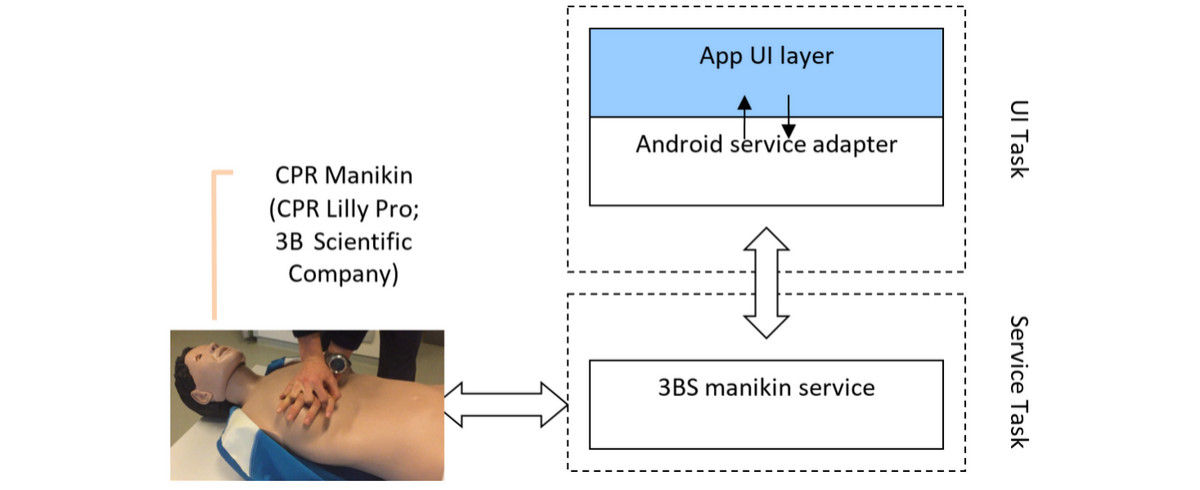
Android App Architecture.

When reviewing a past session, it is also possible to see a detailed timeline of all events in that session, including synchronized BLS actions as shown in Figure 7. Starting a new session triggers the manikin discovery-auto-connect procedure and is possible in 2 different modes. In the “BLS Training” mode, only BLS actions are allowed and a metronome is provided for assistance. The session is not recorded. In the “Full Training” mode, all BLS events are marked along with the BLS algorithm. This mode is intended to be used by the instructor who can mark events that are not electronically tracked to augment the data received from the manikin with their own timestamps. In this mode, the session may be scored and permanently recorded. The app accesses the manikin wirelessly through a secure Bluetooth interface—the BLS Manikin (CPR Lilly; 3B Scientific GmbH). Therefore, the Android device must first be paired with the manikin using the app setup wizard. This wizard also ensures that the app knows which manikin to receive data from. When opening a session, the app creates a Bluetooth connection to the manikin using the serial port profile model. This connection is bidirectional and allows the app to control various functions of the manikin (such as eyes or simulated heartbeat) while also receiving data from it. All of this communication is delegated to a manikin service that the app installs to the system (Figure 8).

Before recording, the app first clears the internal buffers of the manikin service for a new session and then signals the receipt of data. During the session, a steady stream of sensor readings is sent by the manikin to the manikin service. These readings are tracked linearly in order to detect rising and falling edges, which are used to deduce compression and ventilation events. These events are collected into data buffers, which are then transmitted to the Android app. The Android app filters the generated events for possible misdetections and augments them with additional sensor data present in the stream. Such sensor data are received momentarily, but linearly averaged by the app over a window around the event times. The detected and augmented BLS events are then added to the session along with their timestamps and evaluated when the session is over.

### Statistical Analysis

The normality of continuous variables was investigated by Shapiro–Wilk test. Descriptive statistics were presented using mean and standard deviation, median, and range. For comparison of 2 non-normally distributed groups Mann–Whitney *U* test was used. Spearman rho correlation test was used for the correlation between 2 continuous variables that are independent and normally distributed. Statistical signiﬁcance was accepted when 2-sided *P* value was lower than .05. Statistical analysis was performed using the MedCalc Statistical Software version 12.7.7 (MedCalc Software Ltd) [28].

## Results

To be successful from the serious gaming module, participants had to score 80/100 or above. Participants got 80/100 and above in an average of 1.4 (SD 0.6) trials.

The average BLS serious game score was 88.3/100 (SD 5.1), hands-on average score was 70.7/100 (SD 17.3), and the OSCE average score was 84.4/100 (SD 12.9), as shown in Table 2.

**Figure 9:**
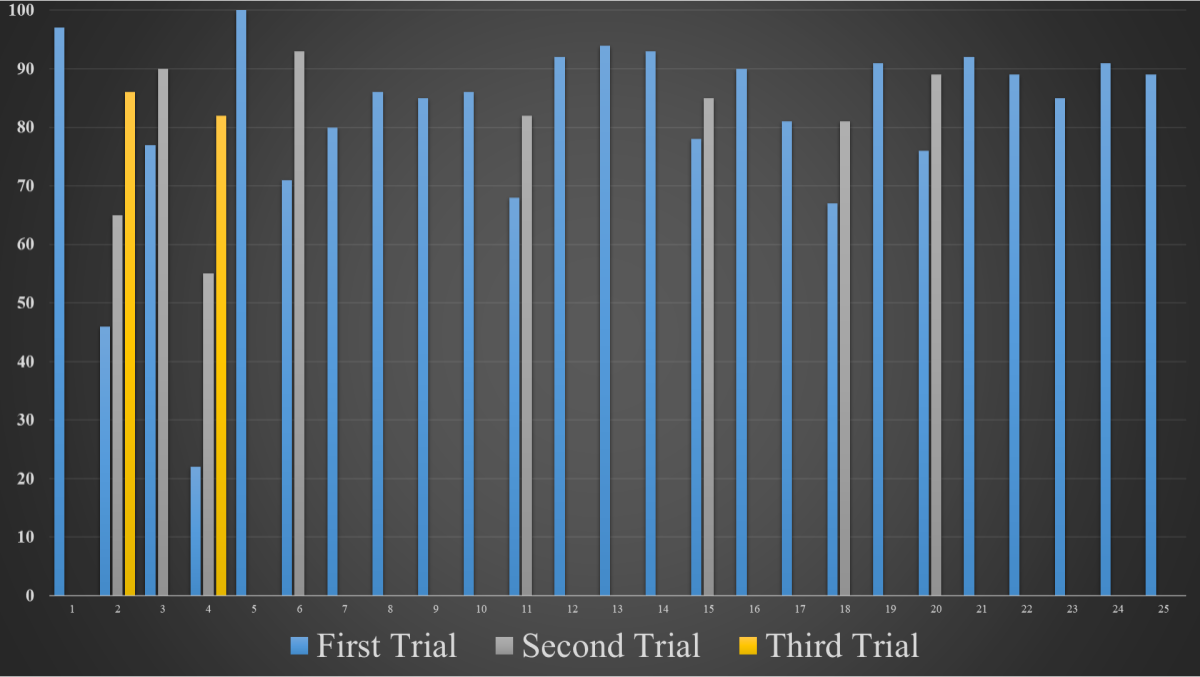
Serious game scores und number of trials of 25 participants.

Serious game scores and number of trials of 25 participants are shown in Figure 9.

**Table 2:** Gender distribution and BLS^a^ serious game, hands-on, and OSCE^b^ scores.

Gender and scores in modules		Values
**Gender**		
	Male, n (%)	11 (44)
	Female, n (%)	14 (56)
**Success on which trial (80 and above)**		
	1, n (%)	17 (68)
	2, n (%)	6 (24)
	3, n (%)	2 (8)
**BLS Serious Game first trial**		
	Mean (SD)	81 (16.9)
	Median (range)	86 (22-100)
**BLS Serious Game second trial**		
	Mean (SD)	80 (13.2)
	Median (range)	83.5 (55-93)
**BLS Serious Game third trial**		
	Mean (SD)	84 (2.8)
	Median (range)	84 (82-86)
**Success on the serious game trial (80 or above was required for success)**		
	Mean (SD)	1.4 (0.6)
	Median (range)	1 (1-3)
**BLS Serious Game best score**		
	Mean (SD)	88.3 (5.1)
	Median (range)	89 (80-100)
**Hands-on app**		
	Mean (SD)	70.7 (17.3)
	Median (range)	73 (37-95)
**OSCE**		
	Mean (SD)	84.4 (12.9)
	Median (range)	89 (55-100)

^a^BLS: basic life support.

^b^OSCE: Objective Structured Clinical Examination.

The score for being successful from the serious gaming module is 80/100 or above. Participants scored 80/100 and above in an average of 1.4 (SD 0.6) trials.

The average BLS serious game score was 88.3/100 (SD 5.1), hands-on average score was 70.7/100 (SD 17.3), and the OSCE average score was 84.4/100 (SD 12.9) as shown in Table 3.

**Table 3:** Comparisons according to gender^a^.

Score comparison		Male	Female	*P*-value
**Success on trial (80 and above)**				.936
	Mean (SD)	1.3 (0.5)	1.4 (0.7)	
	Median (range)	1 (1-2)	1 (1-3)	
**Tablet**				.267
	Mean (SD)	89.8 (6)	87.2 (4.2)	
	Median (range)	90 (81-100)	87.5 (80-93)	
**Hands-on**				.166
	Mean (SD)	76.2 (17.1)	66.4 (16.9)	
	Median (range)	77 (37-95)	70 (37-90)	
**OSCE^b^**				.166
	Mean (SD)	81.2 (12.3)	86.8 (13.3)	
	Median (range)	84 (55-100)	90 (55-100)	

^a^By Mann–Whitney *U* test.

^b^OSCE: Objective Structured Clinical Examination.

According to gender, there was no statistically significant difference in terms of success on any of the trials: tablet, hands-on, and OSCE distributions (Mann–Whitney *U* test, *P*>.05) as seen in Table 3.

**Figure 10:**
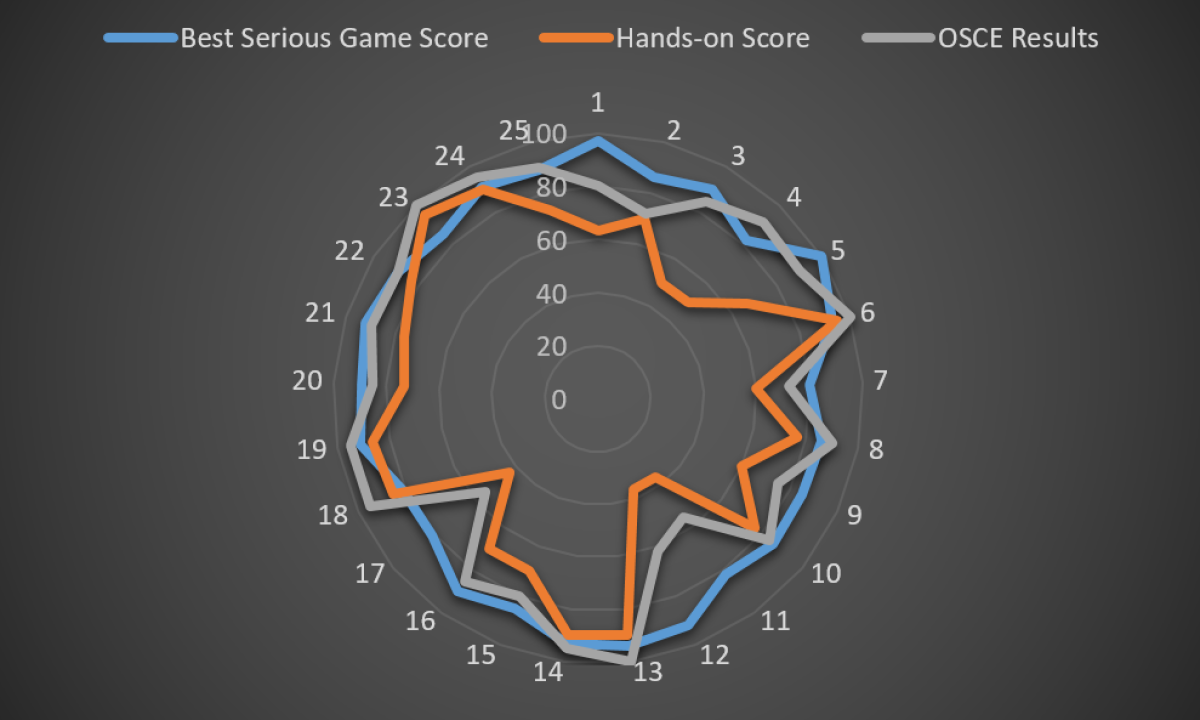
Serious game, hands-on training app and OSCE scores of 25 participants.

As shown in Table 4, there was no statistically significant correlation between success on any trial (score ≥80), serious game, hands-on training app, and OSCE (Spearman rho test, *P*>.05). Serious game, hands-on training app, and OSCE scores of 25 participants are shown in Figure 10.

**Table 4:** Correlation analysis of serious game, hands-on training app, and OSCE scores^a^.

Correlation		Success on which trial (score ≥80)	Tablet	Hands-on	OSCE^b^
**Success on which trial (score ≥80)**					
	*r*	1.000	–0.319	–0.196	0.050
	*P*		.120	.347	.811
**Tablet**					
	*r*	–0.319	1.000	0.319	0.095
	*P*	.120		.120	.651
**Hands-on**					
	*r*	–0.196	0.319	1.000	–0.052
	*P*	.347	.120		.806
**OSCE**					
	*r*	0.050	0.095	–0.052	1.000
	*P*	.811	.651	.806	

^a^By Spearman rho test.

^b^OSCE: Objective Structured Clinical Examination.

## Discussion

The advantages of using serious gaming versus classroom lectures for BLS trainings as hands-on course preparation tool have been demonstrated in several studies [29-31]. There is no commercially available platform combining serious game performance data with hands-on training data obtained by visual checks based on OSCE criteria and sensor data such as compression depth, compression frequency, and ventilation volume from a BLS manikin via Bluetooth before this study. By storing these data of trainees at an individual level in the same LMS, educators were able to see the progress of the trainings and had precise data about compression depth, compression frequency, and ventilation volume retrieved from the manikin’s sensors. In conventional OSCE-based BLS training examinations, educators have to estimate compression depth, compression frequency, and ventilation volume depending on their visual estimation. By using the new platform combining BLS serious game scores and hands-on training data obtained with visual OSCE criteria and sensor data, assessment of the knowledge domain and practical domain could be carried out with performance data that can be retrieved from the LMS at an individual level. Statistical analysis of this study revealed that there was no statistically significant difference in terms of success in any of the trials, (serious game, hands-on, and OSCE) and their score distributions (Mann–Whitney *U* test, *P*>.05) according to gender as shown in Table 3. All participants were given unlimited access for using the serious game module and all of them could reach 80/100 scores or above in an average of 1.4 (SD 0.6) trials. These data revealed that the knowledge domain could be assessed with the examination mode of the serious gaming module. By contrast, no statistically significant correlation was detected between the participants’ serious game, hands-on training app, and OSCE scores (Spearman rho test, *P*>.05) as shown in Table 4. The mean BLS serious game score of the participants was 88.3 (SD 5.1), whereas their mean hands-on training app score was 70.7 (SD 17.3) and OSCE score was 84.4 (12.9). Although identical scoring criteria were used for hands-on training app and OSCE, OSCE scores were 17% higher than hands-on training app scores. After analyzing the difference in scores between the hands-on training app and OSCE, it was found out that these differences originated from scoring parameters such as compression depth, compression frequency, and ventilation volume. These data support the hypothesis of this study that evaluation of BLS trainings would be more objective if these evaluations were carried out with the modality, which combines OSCE scoring criteria with sensor data retrieved from the simulator’s sensors.

### Limitations

Only 1 of the 25 participants (4%) experienced login issues during authorization and due to internet connectivity issues. Two software bugs were encountered during the implementation phase and were all solved in the third version of the serious gaming software. The other problem was a Bluetooth connectivity issue between the hands-on app and BLS manikin in 4 of the 25 (16%) participants. This problem was resolved by removing other Bluetooth devices from the training area. The limitation of this study was that the number of participants was limited to 25. This was due to the COVID-19 pandemic, as the number of students should be limited and diluted in order to minimize any contamination risk.

### Conclusions

The advantages of using serious games before simulation sessions has been proven by several studies. This study revealed that using the platform combining serious game performance data with hands-on training data obtained by combining visual OSCE criteria and sensor data such as compression depth, compression frequency, and ventilation volume from the BLS manikin via Bluetooth enables educators to have an overview of learners’ serious game and hands-on performance data. The platform provides more accurate data in comparison with visual observation when assessing compression depth, compression frequency, and ventilation volume compared with conventional OSCE examinations. This platform is planned to be used with more learners and educators in the upcoming studies. A new augmented virtuality–based module, which will provide mixed reality–based BLS training, is being developed at our center. This new module will be replacing the hands-on training app in our upcoming studies. Using this module, tracking the manikin in virtual space, having haptic response from the manikin, and retrieving its sensor data will be possible. Similar studies are planned by using the mixed reality–based platform in the upcoming months, when the system is ready for use.

## Abbreviations

BLS: basic life support
LMS: learning management system
LRS: learning record store
SCORM: Sharable Content Object Reference Model
xAPI: experience API
